# A Preliminary Investigation of Going Online for Suicide-Related Reasons: The Impact on Suicide Risk and Prevention

**DOI:** 10.3390/bs16091507

**Published:** 2026-08-27

**Authors:** Andrea Lamont-Mills, Amy Briggs, Luke Bayliss, Renée L. Parsons-Smith, Steven Christensen, Peter C. Terry

**Affiliations:** 1 School of Health, Psychological and Medical Sciences, University of Southern Queensland, 11 Sailsbury Road, Ipswich, QLD 4305, Australia; amybriggs81@hotmail.com (A.B.); l.bayliss@griffith.edu.au (L.B.); renee.parsons-smith@unisq.edu.au (R.L.P.-S.); sachristensen41@gmail.com (S.C.); peter.terry@unisq.edu.au (P.C.T.); 2 Institute for Health Research, University of Southern Queensland, 37 Sinnathamby Boulevard, Springfield Central, QLD 4300, Australia; 3 Education, Behavioural and Social Sciences, Adelaide University Online, 259 North Terrace, Adelaide, SA 5000, Australia; 4 National Sports Institute, Bukit Jalil, Kuala Lumpur 57000, Malaysia

**Keywords:** internet, social media, suicide, safety, mood

## Abstract

Online spaces can be a source of suicide safety as well as suicide risk. What potentially restricts their safety value is the limited contemporary evidence regarding who engages online for suicide-related reasons. This study explored characteristic differences between individuals using social media/internet for suicide-related reasons (users) and those who do not (non-users). It sought to identify whether users differ in relation to psychological, interpersonal, and social characteristics compared to non-users. These characteristics were suicide risk (SBQ-R), depression symptomology (PHQ-9), social anxiety (SIAS), perceived social support (MSPSS), help-seeking intentions (GHSQ), mood states (BRUMS), social connectedness (SCS-R), and interpersonal needs (INQ). Ninety adults (*M*age = 42.65, range 18–82 years) at elevated suicide risk (SBQ-R score ≥ 7) and recent experience of suicidal thoughts/behaviors completed an anonymous online survey. Users reported higher overall suicide risk, higher likelihood of disclosing suicidal thoughts, higher levels of depressive symptomology and social anxiety, and more frequent suicidal thoughts than non-users. There was no group difference in the likelihood of a future suicide attempt. Mood state differences indicated users reported higher scores for depression and confusion, with both groups reporting mood profiles associated with psychopathology risk. Across both groups, mood scores predicted 34.1% of the variance in overall suicide risk and 24.0% of the variance in likelihood of attempting suicide in the future. Given the relatively small sample size, the nonsupport of some hypotheses may be due to low power rather than no differences existing. However, the findings provide tentative support for the notion that suicide-related online use may help reduce the self-reported likelihood of future suicide attempts.

## 1. Introduction

### 1.1. Background

Engaging with professional health care can alleviate suicide risk (Øien-Ødegaard et al., 2024). However, individuals at elevated risk (at-risk) do not always reach out and engage with professional help services due to help-seeking barriers such as stigma, financial costs, and excessive wait times (Han et al., 2018; Jafari et al., 2024). The potential of online support services to overcome these barriers is recognized by the World Health Organization (2022) and the Australian Government (2021), with both advocating for formal (i.e., professional) online support services. Despite this, many at-risk individuals prefer informal rather than formal online support (Bell et al., 2018).

Informal online mental health support typically includes peers with lived experience interacting with those seeking help (Rayland & Andrews, 2023). It is convenient, immediate, and offers 24/7 access to anonymous non-professional support. It enables access to free mental health support to individuals who are unable to utilize, or do not wish to reach out to traditional face-to-face professional mental health services (Naslund et al., 2020). The anonymity of these services is highly valued, particularly for those with lived experience of suicide, because it enables the freedom to express suicidal thoughts/behaviors (STBs) openly (Mokkenstorm et al., 2020).

This engagement between individuals with unique yet relatable lived experiences can provide users with a sense of belonging that may be absent in other areas of their lives (Lamont-Mills et al., 2022). Belonging (or lack thereof) and connectedness are key protective factors in contemporary theoretical models of suicide (Joiner, 2005; Klonsky et al., 2021; O’Connor & Kirtley, 2018). It is through this sense of belonging and connectedness that online spaces have been found to reduce the risk of suicide (Choi & Noh, 2020). Informal online support services obviate oft-cited help-seeking barriers and therefore provide an avenue of support and safety for at-risk individuals.

There are concerns about individuals’ safety when going online for suicide-related reasons. This is particularly so for pro-choice or pro-suicide spaces that are usually unmoderated, where users can talk freely about suicide, suicide plans, and the effectiveness of suicide methods (Biddle et al., 2018). Concerns include how increased exposure to suicide-related material and normalizing suicidal behavior potentially facilitates future suicide attempts (Rodway et al., 2022). With concerns come benefits, which include peer support, increased connectedness with others, lowered levels of distress, and a space to express STBs without fear of being judged (Marchant et al., 2017).

Further investigation of individuals who use the internet or social media for suicide-related purposes would be advantageous. For example, it is necessary to understand the characteristics of those who do (users) and do not use (non-users) online spaces to explore how online spaces could be improved to ensure the safety of users and thus potentially prevent suicide. Moderators working in online spaces report feeling overwhelmed due to the high level of uncertainty associated with such spaces, particularly surrounding who is using these spaces to talk about STBs (Perry et al., 2022). Knowing more about who uses these spaces and why is likely to enable better moderator engagement with those at risk.

### 1.2. Existing Research

Few studies have explored and compared characteristics of users and non-users. Harris et al. (2009) compared suicide-related internet users and non-users aged 18 to 71 years from 27 countries. Users (*n* = 165) reported higher levels of depression, suicidal ideation, overall suicide risk, and a greater likelihood of a future suicide attempt than non-users (*n* = 125). Further, users reported lower perceived family support and were more likely to seek help from online sources and less likely to seek help from offline sources than non-users. Mok et al. (2016) compared internet users to non-users using an Australian university sample aged 18 to 24 years and introduced social anxiety as a potential differentiator. Like Harris et al. (2009), they found users had higher levels of suicidal ideation and a greater likelihood of a future suicide attempt than non-users. Users also had higher levels of social anxiety and were more likely to seek help from online forums. However, unlike Harris et al., no differences were found for depressive symptoms despite the elevated suicide risk.

Two studies have replicated the Mok et al. (2016) study, both comprised of participants aged 18 to 24 years with living experience of STBs. Niederkrotenthaler et al.’s (2017) findings were similar to those of Harris et al. (2009) and Mok et al. (2016), with users reporting higher past-year suicidal ideation, a greater likelihood of a future suicide attempt, and being more likely to disclose suicidal thoughts to others. Users also reported lower perceived family support and higher levels of depression. Unlike Mok et al. (2016), there were no differences for social anxiety or lifetime suicidal ideation. The Bell et al. (2018) findings were consistent with Mok et al. (2016), with users reporting higher levels of social anxiety and overall suicide risk than non-users, but there was no difference regarding depressive symptomology. Bell et al. (2018) suggested that mood may have affected participant responses in that responding to surveys in a negative/low mood may have influenced depressive symptomology and therefore recommended assessing mood states in future research. A more recent study looking at online and offline help-seeking behaviors in youths aged 15 to 24 years also reported similar results regarding suicide risk. Wong et al. (2021) found users reported higher levels of suicidal ideation and were more likely to have attempted suicide and sought help from online rather than offline sources than non-users.

### 1.3. Limitations and Gaps of Existing Research

Whilst there are consistent (e.g., elevated suicide risk) yet different (e.g., perceived social support) findings, limitations regarding age and the singular focus on internet use in this past research are problematic. This focus on just internet use (i.e., websites/forums) overlooks simultaneous suicide-related internet and social media (i.e., apps) use (Dutton & Reisdorf, 2019). Further, most previous studies recruited university students (see Bell et al., 2018; Mok et al., 2016; Niederkrotenthaler et al., 2017). The population most likely to use the internet for suicide-related reasons, general mental health and/or suicide-specific websites/forums users, was not specifically sampled. This inadvertently may have excluded the very people who use the internet from the very research that aimed to help them. Therefore, user characteristics associated with a broader range of individuals who simultaneously use the internet and social media for suicide-related reasons remain unclear. Further, most user characteristic research was conducted several years ago, and there has been rapid growth in usage of the internet and social media across all age groups, particularly in Australia (Australian Communications and Media Authority, 2022). User characteristics findings from 2009, 2016, 2017, and 2018 may now be outdated. Despite the potential for informal online spaces to play a critical role in suicide prevention (Sweeney et al., 2024), repeatedly using samples comprising younger individuals and their experiences restricts the potential of these spaces in helping a broader age range of individuals.

There is increasing recognition that online spaces can be places for suicide safety, not just harm. What potentially restricts their safety value is the limited contemporary evidence regarding who engages online for suicide-related reasons. Not only is existing research somewhat dated now, but it is also predominantly focused on young adult and adolescent samples, has used a narrow range of characteristics, and has typically conceptualized online suicide-related use as a single behavior (i.e., internet or social media). How this existing evidence generalizes beyond younger, single-behavior use remains unclear. Critical gaps exist in terms of the experiences and characteristics of middle-aged-to-older adults, broader psychological, interpersonal, and social characteristics, and the characteristics of individuals who engage with multiple online suicide related-resources. Thus, it is necessary to understand the characteristics of those who go online for suicide-related reasons beyond these earlier studies.

### 1.4. Study Justification, Research Questions and Hypotheses

The present study was a partial replication of Harris et al. (2009), Mok et al. (2016), Bell et al. (2018), and Niederkrotenthaler et al. (2017). We extended this past research in four ways. First, the sample only included individuals with recent STBs (i.e., less than six months) because of the increased risk of suicide attempt/re-attempt within this period (Demesmaeker et al., 2021). Evidence suggests that individuals at elevated risk of suicide are more likely to seek help (Mok et al., 2021). Focusing on recent STBs aimed to limit the possibility that any observed group differences are reflective of general help-seeking intentions rather than differences in user/non-user characteristics. Second, there was no maximum age restriction on participants, although 18 years was maintained as the minimum age. Third, both internet and social media suicide-related use were included rather than a single medium to reflect contemporary use of online spaces for help-seeking purposes. Previous research limited online use to either social media or Internet use. However, emerging evidence indicates that people often seek help from both the Internet and social media, with each serving different informational purposes (Stifjell et al., 2025). Restricting use to one medium is unlikely to capture the complex ways those with a lived experience of suicide engage in online spaces. As the present study aimed to examine differences between individuals who engage online for suicide related purposes and those who do not, the inclusion of both social media and the Internet enabled a focus on characteristic differences rather than online platform preference. Finally, mood and interpersonal needs measures were added to reflect Bell et al.’s (2018) suggestion for future research and contemporary suicide theories, respectively.

The aim of this study was to explore differences in characteristics between individuals who go online for suicide-related reasons and those who do not. It sought to identify whether individuals who go online for suicide-related reasons differ in relation to psychological, interpersonal, and social characteristics compared to those who do not. These characteristics were suicide risk, social anxiety, depression symptomology, social connectedness, help-seeking intentions, sources of perceived social support, interpersonal needs, and mood states. The research hypotheses (H) were:

**H1.**  *Users would report more suicidal ideation and/or attempts, frequency of past year suicidal ideation, disclosure of suicidal intent to others, likelihood of future suicidal attempts, and total suicide risk than non-users (Bell et al., 2018; Harris et al., 2009; Mok et al., 2016; Niederkrotenthaler et al., 2017).*

**H2.**  *Users would report higher levels of social anxiety, depression, and social connectedness than non-users (Bell et al., 2018; Harris et al., 2009; Mok et al., 2016; Niederkrotenthaler et al., 2017).*

**H3.**  *Users would be more likely to seek help from informal and online sources but less likely to seek help from formal and offline sources than non-users (Harris et al., 2009; Mok et al., 2016; Wong et al., 2021).*

**H4.**  *Users would report lower levels of significant others, family, and friends’ perceived social support (Harris et al., 2009; Niederkrotenthaler et al., 2017).*

**H5.**  *Users would report higher levels of perceived burdensomeness and thwarted belongingness (Ladis et al., 2024).*

**H6.**  *Users would score higher on negative mood dimensions associated with suicide ideation, notably depressed mood and confusion (Dickhoff et al., 2026; Kneeland et al., 2025).*

## 2. Materials and Methods

### 2.1. Sample

**Figure 1:**
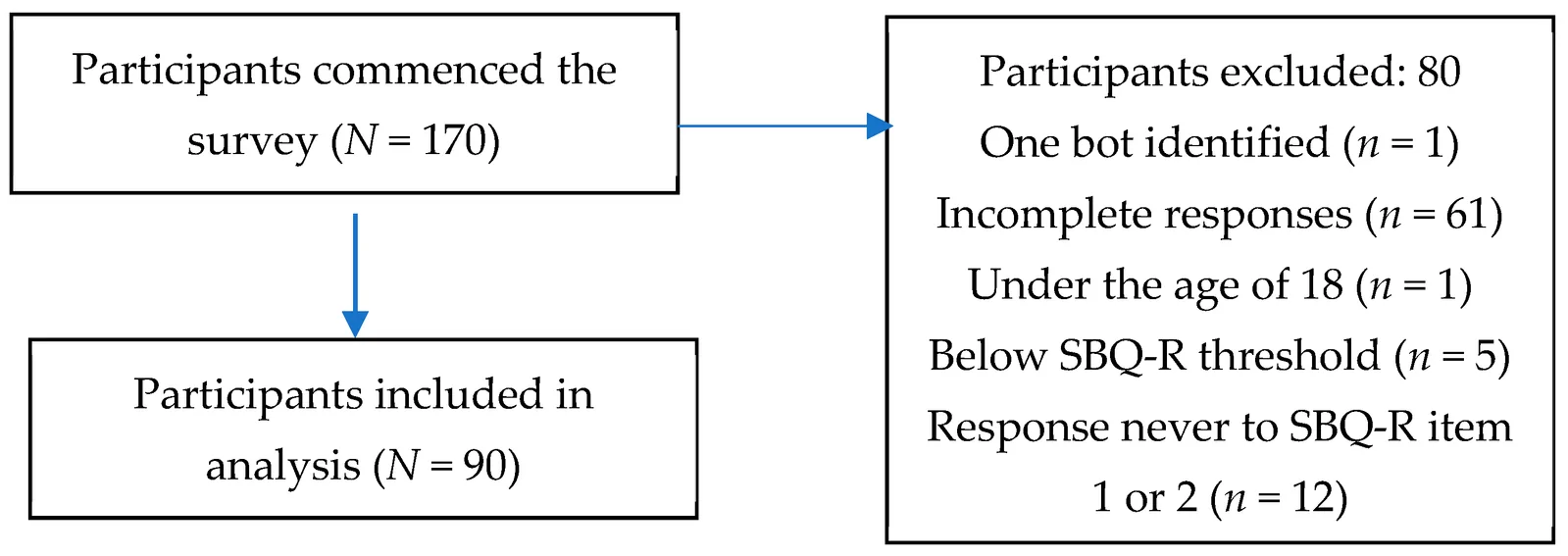
Data screening process.

A total of 170 participants completed or began the survey. After data screening (see Figure 1), 90 participant responses were included for analysis (see Table 1). Of these, 33 were users based on their responses to the following questions: “In the past 6 months, have you used the internet and/or social media for suicide related purposes? Suicide-related purposes is going online for reasons relating to your own feelings of suicide, looking for information or communicating with others anonymously online”, and “When you used the internet and/or social media for suicide related purposes was it … To ONLY find offline sources of help/To communicate with offline friends ONLY (e.g., Facebook)/ONLY briefly as I did not find it helpful/I used it for OTHER reasons than the above (e.g., information or communicating with anonymous online people whose real identity I did not know”. The inclusion of an operational definition of suicide-related purposes was to ensure that participants had a standardized understanding of this construct.

### 2.2. Measures

The Suicide Behaviors Questionnaire-Revised (SBQ-R; Osman et al., 2001) measures suicide risk and consists of four items, each with a different rating and response scale. Items can be scored separately and analyzed independently to assess different dimensions of suicide risk, with higher scores indicating higher suicide dimension risk. A total suicide risk score can also be obtained by calculating the sum of responses to each item, with total scores ranging from 3 to 18. A total score of 7 or above indicates elevated suicide risk. Internal consistency for the SBQ-R and other measures is displayed in Table 2.

Social anxiety was measured using the 20-item Social Interaction Anxiety Scale (SIAS; Mattick & Clarke, 1998). A total score is obtained by summing responses to all 20 items, with scores ranging from 0 to 80. Higher scores indicate higher social anxiety.

The nine-item Patient Health Questionnaire (PHQ-9; Spitzer et al., 1999) was used to measure depression as it also has diagnostic validity (Sun et al., 2020). To get a total score, responses to the nine items are summed, and scores range between 0 and 27. Mild depression is indicated by scores that fall between 5 and 9, moderate 10–14, moderately severe 15–19, and severe 20–27.

The Social Connectedness Scale-Revised (SCS-R; Lee & Robbins, 1995) was used to measure the degree to which participants feel a sense of belongingness and closeness to others within their social environment. Responses to all 20 items are summed to get a total score ranging from 20 to 120. An item mean score is also calculated by dividing the total score by the number of scale items, with item mean scores ranging from 1 to 6. A mean item score of less than 3.5 indicates potential disconnection from the participants’ social environment, with higher scores indicating a stronger sense of belongingness and closeness to others.

The General Help-Seeking Questionnaire (GHSQ; Wilson et al., 2005) comprises a single question measuring help-seeking intentions for suicidal thoughts from 15 sources. Items relating to formal (e.g., doctor), informal (e.g., friend), online (e.g., online forum), and offline (e.g., mental health professional) sources were added together to compare across these groupings. Scores for each group could range from 1 to 7.

Social support was measured using the Multidimensional Scale of Perceived Social Support (MSPSS: Zimet et al., 1988). It is a 12-item self-report scale comprising three subscales (significant other, family, friend). To enable comparison with Bell et al. (2018) and to be consistent with the developers’ intentions for use, mean subscale scores were calculated by summing the responses within each subscale and dividing by 4. Scores less than 3 indicate low support from that source.

The 15-item Interpersonal Needs Questionnaire (INQ; K. A. Van Orden et al., 2012) measured perceived burdensomeness (PB) and thwarted belongingness (TB). The questionnaire comprises two subscales, which include six PB items and nine TB items. To obtain subscale scores, items within each subscale are summed and range from 6 to 42 for PB and 9 to 63 for TB. Higher scores are indicative of higher PB and TB. A PB score of 17 and above and/or a TB score of 35 and above are indicative of elevated suicide risk.

The Brunel Mood Scale was used as a brief measure of mood state (Terry et al., 2003). The 24-item scale consists of six subscales (i.e., tension, depression, anger, vigor, fatigue, confusion), each comprising four items. Subscale raw scores range from 0 to 16, with higher scores indicative of higher levels on each of the mood dimensions (e.g., depression). Raw scores for each of the six subscales were converted into T-scores using normative data (Terry & Parsons-Smith, 2021). Subscale T-scores can be plotted graphically to display an overall mood profile (e.g., inverse iceberg profile; Parsons-Smith et al., 2017; Terry & Parsons-Smith, 2021).

### 2.3. Data Analysis

Data were screened for potential internet bot responses as per Storozuk et al.’s (2020) recommendations related to time and speed of survey completion and response inconsistencies (i.e., nonsensical or contradictory responses). One suspected bot response was removed from analyses. All analyses were conducted using SPSS for Windows, Version 28, IBM Corporation, Armonk, NY, USA (IBM Corp, 2021). Between-group differences for most measures were assessed using Mann-Whitney U Tests as the small sample size, resultant low power, and normality assumption issues rendered parametric procedures inappropriate (Fein et al., 2022). Mann-Whitney U Tests are designed for analyzing small samples and remain usable with unequal-sized groups (Chicco et al., 2025).

Mood scores were shown to be normally distributed, with no significant skewness or kurtosis. A single-factor MANOVA was used to compare the mood profiles of user and non-user groups, which is robust to unequal sample sizes up to a ratio of 2:1 (Tabachnick & Fidell, 2019). A power analysis conducted using the Stats Kingdom calculator (https://www.statskingdom.com/sample_size_manova.html (accessed on 18 January 2026)) indicated that a sample of 68 was required when using a one-way MANOVA to identify medium effects (0.30) with 80% power at *p* < 0.05. However, given the six mood dimensions in the analysis and to guard against Type I errors, a Bonferroni adjustment was made to the alpha level for the univariate analyses (0.05/6, *p* = 0.008). Group means for the six subscale raw scores were converted into subscale T-scores with reference to population norms and plotted to visually represent mood scores and compared against normative mood profiles (Terry & Parsons-Smith, 2021). Multiple linear regression was used to determine the extent to which mood scores could predict suicide risk.

### 2.4. Procedure

Interested participants clicked on the survey link or scanned the QR code on the recruitment flyer to access the online survey, where the first screen was the Participant Information Sheet (PIS). The PIS outlined details of the project, with consent being explicitly gained from participants by clicking the “consent” box at the bottom of the PIS. Participants were unable to begin the survey unless they did this.

Once consent was gained, participants were presented with demographic questions followed by the measures presented above. Participants were given the option of completing the survey anonymously or providing their contact details should they later wish to withdraw their participation or wanted to stop and return to the survey at a later stage. Consistent with trauma-informed research participation, participants were able to not answer and skip questions, giving participants agency over how and what questions they responded to. Participants who scored high on the SBQ-R (i.e., ≥7) were automatically alerted to this after survey submission, as this is indicative of heightened suicide risk, and encouraged to contact one of the crisis-support contacts listed on the last screen.

## 3. Results

Of the 27 suicide-related users, 5 (18.5%) reported using the Internet only, 3 (11.1%) reported using social media only, with the majority, 19 (70.4%), using both the Internet and social media for suicide-related reasons. Suicide-related users were also asked about the type of sites (e.g., pro-life/anti-suicide) they had used. Twenty-six participants responded, with nine (33.3%) reporting they had used pro-life/anti-suicide sites, five (18.5%) reporting using pro-suicide sites, and the majority, 12 (44.4%), reporting that they had used both.

Results provided partial support for H1 (see Table 3), with users reporting significantly higher overall suicide risk, higher frequency of past year suicidal ideation, and being more likely to disclose suicidal thoughts to others than non-users. There were no significant differences between users and non-users for history of suicidal ideation or likelihood of future suicidal attempts. H2was supported, with users reporting significantly higher levels of social anxiety and depression than non-users, and non-users reporting significantly greater social connectedness than users. No significant differences between users and non-users were found on the characteristics associated with H3, H4, and H5.

Given the number of planned comparisons conducted, a Holm-Bonferroni correction was undertaken as a sensitivity analysis and to assess the robustness of findings. Based on the correction, four significant findings remained: users reported higher overall suicide risk, past year suicidal ideation, social anxiety, and depression when compared to non-users. The effect sizes associated with these adjusted significant findings are small (SBQ-R Frequency) or small-to-medium (SBQ-R Total, PHQ-9, SIAS). The effect sizes relating to the two remaining significant findings at the unadjusted level (i.e., users being more likely to disclose suicidal thoughts to others and reporting lower levels of social connectedness when compared to non-users) are also small; therefore, these unadjusted findings need to be interpreted with caution.

Regarding mood, the multivariate statistic did not quite reach significance (*p* = 0.08), although group membership was shown to explain 12.4% of the variance in mood scores. Univariate follow-ups are usually only warranted if the omnibus statistic is significant. However, univariate group differences in BRUMS subscale scores were considered from an exploratory perspective using a cautious approach, with a Bonferroni-adjusted alpha level of 0.008 being adopted. Users reported significantly higher levels of depression and confusion than non-users, with moderate effect sizes (Table 4). They also reported higher levels of anger, fatigue, and tension, and lower levels of vigor, with small-to-moderate effect sizes, although these differences were non-significant.

Important findings emerged related to the predictive effectiveness of mood scores on suicide risk. Across the whole sample, mood profiles were shown to predict 34.1% of the variance in overall suicide risk (Adj. R^2^ = 0.341, F = 8.66, *p* < 0.001) with depressed mood scores contributing most to the predictive model (Standardized β = 0.457, *p* < 0.01). Similarly, mood profiles predicted 24.0% of the variance in likelihood of attempting suicide in the future (Adj. R^2^ = 0.240, F = 5.70, *p* < 0.001), with depressed mood scores again being the best single predictor (Standardized β = 0.564, *p* < 0.01).

**Figure 2:**
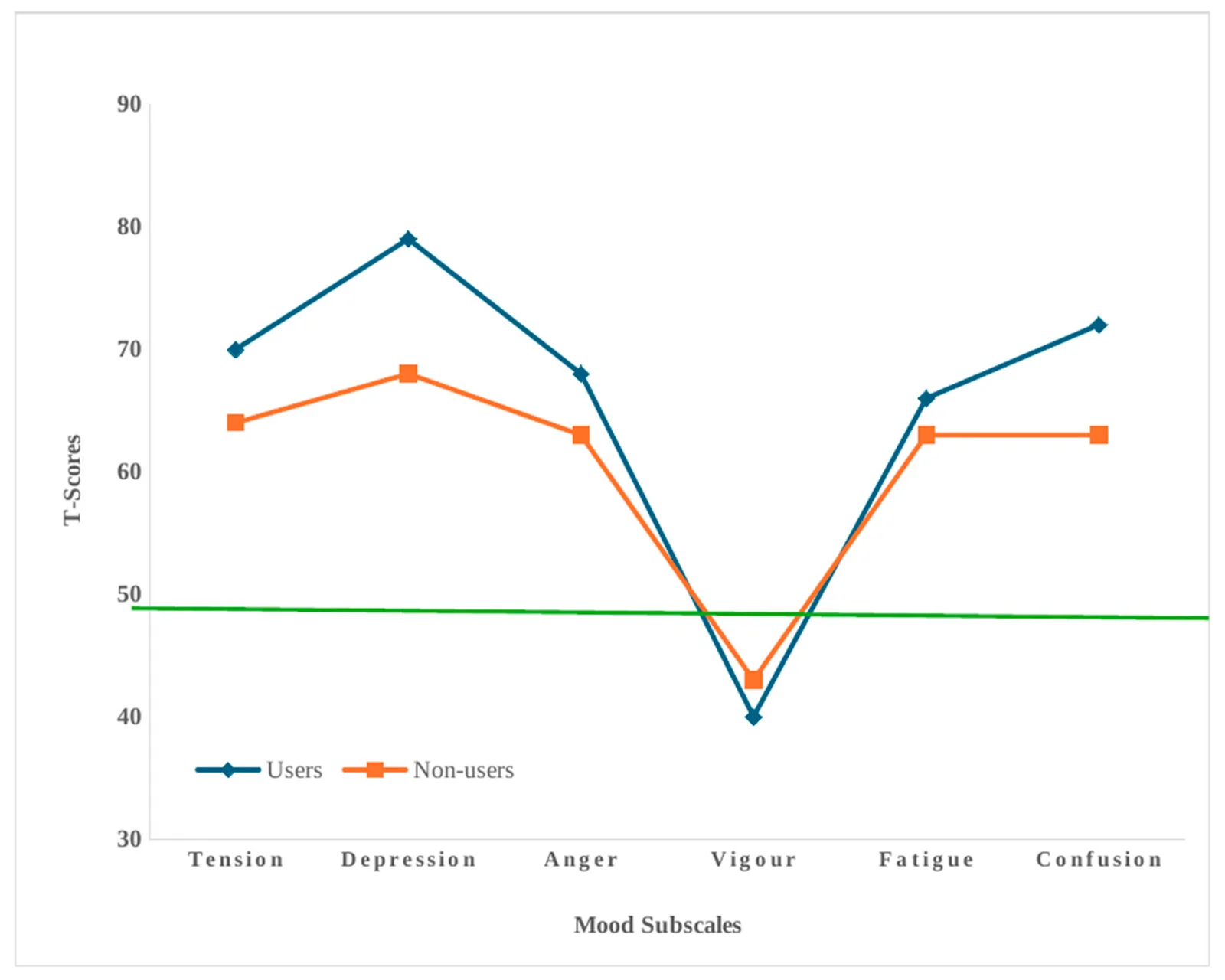
Mood profile for user and non-user plotted against population norms (green line).

Our between-group findings regarding mood were also noteworthy. Both the user and non-user groups reported very negative mood profiles compared to population norms (see Figure 2). Mean mood scores for the user group approximated an inverse Everest profile (Parsons-Smith et al., 2017; Terry & Parsons-Smith, 2021) with very high scores (≥2 standard deviations above the mean) for tension, depression, and confusion, high scores (≥1 standard deviation above the mean) for anger and fatigue, and low scores (≤1 standard deviations below the mean) for vigor. Typically, such a profile is reported by <5% of the population and is proposed to signal elevated risk of psychopathology (Brandt et al., 2016; Van Wijk et al., 2013). The mean non-user mood scores represented an inverse iceberg profile (Morgan, 1985; Terry & Parsons-Smith, 2021), with high scores (≥1 standard deviation above the mean) for tension, depression, anger, fatigue, and confusion, and low scores (≤1 standard deviations below the mean) for vigor, typically reported by about 12% of the population and also associated with risk of mental ill-health (Morgan, 1985; Raglin, 2001).

## 4. Discussion

This study extends previous research that explored the characteristics of individuals who go online for suicide-related reasons. Whilst some of our findings broadly align with earlier studies, there are also differences. The suicidal ideation frequency and overall suicide risk findings are consistent with previous studies (Bell et al., 2018; Harris et al., 2009; Mok et al., 2016; Niederkrotenthaler et al., 2017). However, findings relating to suicidal ideation history and disclosure are only consistent with Niederkrotenthaler et al. (2017).

Going online for suicide-related reasons typically means users are talking and interacting with others about their suicidal thoughts (Biddle et al., 2020); thus, the very act of going online for such purposes requires disclosure. It is not surprising that users were higher on both frequency and disclosure. Although online behaviors can be medium-specific (Brennan et al., 2022) and disclosure has been found to differ between social networking sites (Jaidka et al., 2018), users in this study potentially had more opportunities to disclose because of their dual online medium use compared to participants who were limited to reporting on only one online medium in other studies. However, the self-disclosure finding needs to be considered cautiously given the number of planned comparisons that were conducted. Further research is therefore needed to see if this finding can be replicated. Given all participants reported recent experiences of STBs, the contrasting findings for suicidal ideation history may reflect participant eligibility criteria. The current study was focused on recent usage and recent STBs experiences, whereas previous studies did not limit usage or STBs experience.

The self-reported likelihood of future suicide attempt findings is noteworthy as it is inconsistent with previous research. A possible explanation is the older average age of participants in this study. This may be because STBs have been found to decrease with age, albeit with deaths by suicide increasing (K. Van Orden & Deming, 2018). Nonetheless, given concerns about potential risks associated with suicide-related online use (Marchant et al., 2017), our findings suggest that going online may not be as risk-inducing as previous research suggests (Biddle et al., 2018; Rodway et al., 2022).

Our data revealed users had higher levels of depression and social anxiety, and lower levels of social connectedness compared to non-users. The depression findings are inconsistent with Mok et al. (2016) and Bell et al. (2018) but consistent with Harris et al. (2009) and Niederkrotenthaler et al. (2017). Given depression is an STB’s risk factor (Orsolini et al., 2020), this may reflect our, Harris et al.’s (2009), and Niederkrotenthaler et al.’s (2017) broader recruitment strategies. Namely, the strategies may have captured individuals also seeking help online for depressive symptoms. Although the depression findings are inconsistent with Mok et al. and Bell et al., our findings regarding social anxiety are consistent with those studies. This is not unexpected given that the anonymity offered by informal online spaces is one of the reasons why individuals go online for suicide-related purposes (Mokkenstorm et al., 2020). For individuals with elevated social anxiety levels, this anonymity may be less anxiety-inducing than offline interactions, as users can communicate more easily with others online, have more control over their self-identity, and avoid unwanted social interactions (Yen et al., 2012). Further, users reported less social connectedness than non-users, despite previous research finding a benefit of going online to be an increased sense of connectedness with others (Robinson et al., 2016). Noting that this finding was not significant when the Holm-Bonferroni adjustment was applied, any comparison to previous research needs to be considered carefully, with more research required to determine if this finding holds true. Given the stigma associated with suicide and the association between stigma and social isolation (Shoib et al., 2022), it may be social isolation rather than social connectedness that is a specific user characteristic.

Consistent with previous studies (Mok et al., 2016; Bell et al., 2018), no significant differences were found between user groups regarding online, offline, formal, and informal sources. Despite this, mean scores for both groups were higher for formal, offline help-seeking sources, suggesting there may be a preference for formal help. This is supported by the high proportion of both users (87.9%) and non-users (82.5%) having previously sought formal mental health support. However, the smaller number currently using mental health support suggests that whilst both user groups may be open to formal support, they may be turning to informal support due to having difficulties accessing formal support, because of cost or wait times, and/or finding that informal sources provide sufficient support.

In relation to social support, there were no significant differences between users and non-users. Our results align with Harris et al. (2009), Mok et al. (2016), Niederkrotenthaler et al. (2017), and Bell et al. (2018), in that no significant group differences were found for the friend and significant other subscales, suggesting that lower social support from these sources may contribute to online use. However, Harris et al. (2009), Niederkrotenthaler et al. (2017) and Bell et al. (2018) reported statistically significant differences in perceived social support from family, with users reporting lower perceived levels. This difference from these two previous studies may be reflective of the older mean age of participants in our study. That is, what constitutes family for our older participant group (e.g., partner/children) may be different from how younger individuals in the Niederkrotenthaler et al. (2017) study constituted family (e.g., siblings/parents), and the difference in results may be reflective of perceived family support sources for our older cohort. Whilst the age range of participants within the Harris et al. (2009) study was similar, the mean age of the two user groups was not reported. Therefore, it is unknown whether the group differences in perceived family support reported in our study may be attributed to a difference in age when compared to the Harris et al. (2009) study.

We did not find significant group interpersonal need differences. Scores for both user groups indicated elevated suicide risk and were higher than scores reported during the original scale validation (see K. A. Van Orden et al., 2012) for adults aged 18 to 35 years, 57 to 93 years, and adults from a clinical sample. The higher scores and elevated suicide risk are perhaps not surprising, given only participants with heightened risk of suicide were included in our sample. The non-significant user group differences may be related to the underlying dimensions of the constructs. That is, the perceived (or actual) reciprocal care users experience online may result in lowered feelings of burdensomeness and increased belongingness. An alternate explanation may be that users have higher levels of hopelessness that is suggested to contribute to an active desire for suicide (Joiner, 2005; Klonsky et al., 2021; O’Connor & Kirtley, 2018). As such, it may be hopelessness that differentiates between those who turn online for help and support and those who do not, rather than burdensomeness and lack of belongingness.

### 4.1. Implications

A notable implication is that going online for suicide-related reasons did not equate to an increase in the self-reported likelihood of attempting suicide in the future and that individuals self-reported that they are disclosing their STBs when going online. Perhaps online spaces are places where users feel safe to talk about their STBs, and ways to facilitate, rather than stymie, such talk are needed. Therefore, organizations offering formal/informal online support for such individuals may like to consider ways in which to facilitate the disclosure users need and how this is best moderated.

Most users used both the internet and social media for suicide-related reasons. Organizations may also like to consider having dual aspects to their online spaces, both internet and social media, given that participants were more likely to use both. Older individuals self-report that they use online spaces for suicide-related reasons; therefore, organizations may like to consider consulting and collaborating with older, not just younger, users in the future development of suicide prevention spaces if not doing so already. This includes any language guidelines for the safe use of such spaces.

The potential predictive effectiveness from the mood profiles is especially noteworthy considering that all participants came from a population with an elevated risk of suicide. Mood scores tended to be elevated well above population norms for tension, depression, anger, fatigue, and confusion, especially among the user group. Collectively, the six mood scores, particularly scores for depressed mood, predicted about one third of total suicide risk and about one quarter of the risk of attempting suicide in the future. It is important not to overstate the clinical relevance of the group differences in mood scores. This finding may be relevant to practitioners working in the area who might use mood profiling to monitor mood disturbances, especially increases in depressed mood scores, that could signal potential elevated risk of a suicide attempt. The more negative moods reported by the user group may be part of the impetus for them going online for suicide-related reasons in the first place.

### 4.2. Future Research

Further research examining user characteristics for individuals who use both the internet and social media is needed with different populations to further support these findings. Future studies using a theoretical approach could include measures to assess hopelessness and social isolation to consider if these factors distinguish users from non-users. The inclusion of measures for online use relating to general mental health may provide insight into whether users’ suicide-related online use is more related to general mental health usage than STBs. Further, given that mood fluctuates (Mirchi et al., 2019) and there is growing recognition of fluctuations in STBs (Bryan et al., 2017; Rogers et al., 2022), the role that mood variability may play in suicide-related online use warrants further examination. Critically, given that some users are using both pro-life and pro-suicide spaces, research focus on pro-suicide space use could assess potential differences between pro-life and pro-suicide users and how users are engaging in these spaces.

This study captured a wide age range of participants, including older populations who are not only most at risk of suicide, but who have previously been overlooked in suicidology research (Schlichthorst et al., 2020). Further, our user vs. non-user group size difference is consistent with Harris et al. (2009), Mok et al. (2016), and Bell et al. (2018). Online use was also broad, capturing pro-life as well as pro-choice/pro-suicide/pro-death site users. Unlike previous studies, we did not seek to restrict what was meant by these terms, leaving it open for participants to determine what type of site they believe they had visited. This was because there is contention regarding what constitutes a pro-death or pro-suicide site, with Till and Niederkrotenthaler (2014) arguing that there is a high degree of variability and subjectivity between such sites. Further, the present study is one of the few to have investigated mood states and their relationship to suicide-related online use.

### 4.3. Limitations

Limitations include low power due to a relatively small sample size, although our sample size was similar in size to previous research in the area (see Bell et al., 2018; Niederkrotenthaler et al., 2017). Nevertheless, some statistical comparisons are acknowledged to be underpowered to detect small-to-moderate group differences even if real differences did exist. This may help explain the null differences for some variables where differences were hypothesized for help-seeking sources, perceived social support, perceived burdensomeness, and thwarted belongingness. The non-significant findings need to be interpreted with caution, as non-significance does not mean that the groups were the same, particularly given sample size and the associated power concerns. The relatively small sample size also raises questions about the reliability of the findings, as they may be reflective of the participants in this study and may differ with a different sample. Future research should focus not only on larger samples but also on approaches that address online recruitment concerns. Furthermore, although analyses were planned and based on theory and past research, there is a possibility of an inflated Type 1 error being present in this study. When the Holm-Bonferroni correction was applied, four of the six findings remained significant.

Although the SBQ-R, BRUMS vigor, and GHSQ informal Cronbach alphas are below the conventional 0.70 criterion (0.67, 0.67, 0.61 respectively), brief scales often demonstrate lower reliability estimates (see Cortina, 1993); as such, the alpha values were judged to be acceptable, but results related to those scales should be interpreted with caution. The survey design lends itself to recall bias, which raises questions about the reliability of findings. However, personally meaningful and salient experiences tend to be more easily and clearly recalled (Neisser & Harsch, 1992), thus potentially mitigating the impact of this bias. Being cross-sectional research also means no causal inference can be made. Whilst we did include an operational definition of suicide-related purposes to standardize participant understanding, this does not remove the possibility of individual differences in interpretation of this concept. A further limitation relates to selection bias associated with the predominantly online recruitment strategy. While this may have attracted participants who were more comfortable engaging in online spaces and seeking help online, the within-group descriptive data indicates a possible preference for offline support. This does not detract from the possibility that individuals who prefer seeking help online may have been over-represented in the study, thus limiting the generalizability of the findings. However, the recruitment strategy included various mental health organizations, suicide prevention networks, social media groups, and community websites to minimize this potential bias.

## 5. Conclusions

Individuals experiencing STBs are increasingly going online for support. Accompanying this increase are concerns that this may elevate their suicide risk. In line with earlier studies, the present study found that users reported experiencing greater depressive symptoms and social anxiety and were more likely to report disclosing their suicidality than non-users. No statistically significant difference was observed between users and non-users in self-reported risk of a future suicide attempt. Whilst noting the correlational nature of the user research conducted thus far, including the current study, our findings and evidence from other user characteristics and benefits of going online for suicide related reasons research provide tentative support for the proposition that going online and talking about STBs may have potential as a protective mechanism to keep individuals safe. As a suicide prevention approach, going online for suicide-related reasons may be helpful for those individuals who find it difficult to receive other forms of support during a heightened state of suicidal desire, although future research is needed to determine if this is the case.

## Figures and Tables

**Table 1:** Full sample demographics.

Characteristic	
Gender ^1^	
Cisgender female	51 (56.7%)
Cisgender male	28 (31.1%)
Other	8 (8.8%)
Did not answer	3 (3.3%)
Prefer not to say	2 (2.2%)
Age range	18–82 years
Employment status	
Full-time	23 (25.6%)
Part-time	15 (16.7%)
Casual	10 (11.1%)
Seeking opportunities	11 (12.2%)
Unable to work	17 (18.9%)
Retired	12 (13.3%)
Did not answer	2 (2.2%)
Ethnic and cultural group	
Australian	63 (70.0%)
Australian Aboriginal or Torres Strait Islander	4 (4.4%)
Asian	4 (4.4%)
European	4 (4.4%)
Other	12 (13.2%)
Did not answer	3 (3.3%)
Marital status	
Single	27 (30.0%)
Never married	5 (5.6%)
Married	31 (34.4%)
De facto	7 (7.8%)
Separated	7 (7.8%)
Divorced	10 (11.1%)
Widowed	1 (1.1%)
Did not answer	2 (2.2%)
Currently seeing MHP ^2^	
Yes	57 (63.3%)
No	31 (34.4%)
Did not answer	2 (2.2%)
Past seen MHP	
Yes	76 (84.4%)
No	12 (13.3%)
Did not answer	2 (2.2%)

^1^ The total number and percentage for gender exceeds 100% as participants were able to select more than one gender. ^2^ MHP = mental health professional.

**Table 2:** Internal consistency reliability coefficient of measures.

Measure	Alpha
Suicide Behaviors Questionnaire-Revised	0.67
Social Interaction Anxiety Scale	0.93
Patient Health Questionnaire	0.92
Social Connectedness Scale-Revised	0.74
General Help-Seeking Questionnaire—informal sources	0.61
General Help-Seeking Questionnaire—formal sources	0.74
General Help-Seeking Questionnaire—offline sources	0.74
General Help-Seeking Questionnaire—online sources	0.90
Multidimensional Scale of Perceived Social Support—family	0.96
Multidimensional Scale of Perceived Social Support—friend	0.94
Multidimensional Scale of Perceived Social Support—significant other	0.96
Interpersonal Needs Questionnaire—perceived burdensomeness	0.92
Interpersonal Needs Questionnaire—thwarted belongingness	0.91
Brunel Mood Scale—anger	0.90
Brunel Mood Scale—confusion	0.85
Brunel Mood Scale—depression	0.95
Brunel Mood Scale—tension	0.80
Brunel Mood Scale—vigor	0.67
Brunel Mood Scale—fatigue	0.91

**Table 3:** Descriptive statistics and results of Mann-Whitney U tests for measures of suicidality, social anxiety, depression, social connectedness, help-seeking intentions, sources of perceived social support, and interpersonal needs.

Sub-Scale	Suicide-Related Users (*n* = 33)	Non-Suicide Related Users (*n* = 57)	Mann-Whitney U Results
SBQ-R History	M = 3.55	M = 3.28	U = 773.50
	SD = 0.56	SD = 0.75	z = −1.55
	Mdn = 4.00	Mdn = 3.00	r = −0.16
SBQ-R Frequency	M = 4.52	M = 3.81	U = 639.50 *
	SD = 0.71	SD = 1.23	z = −2.71
	Mdn = 5.00	Mdn = 4.00	r = −0.29
SBQ-R Disclosure	M = 2.36	M = 1.91	U = 657.00 *
	SD = 0.78	SD = 0.81	z = −2.52
	Mdn = 3.00	Mdn = 2.00	r = −0.27
SBQ-R Future	M = 3.52	M = 3.04	U = 727.50
	SD = 1.06	SD = 1.40	z = −1.84
	Mdn = 4.00	Mdn = 3.00	r = −0.19
SBQ-R Total	M = 13.94	M = 12.04	U = 594.50 *
	SD = 2.05	SD = 3.11	z = −2.92
	Mdn = 14.00	Mdn = 12.00	r = −0.31
SIAS	M = 49.73	M = 36.68	U = 589.50 *
	SD = 14.09	SD = 17.58	z = −2.94
	Mdn = 49.00	Mdn = 36.00	r = −0.31
PHQ-9	M = 17.82	M = 13.21	U = 602.00 *
	SD = 7.46	SD = 6.88	z = −2.84
	Mdn = 19.00	Mdn = 11.00	r = −0.30
SCS-R	M = 2.71	M = 3.12	U = 683.00 *
	SD = 0.73	SD = 0.86	z = −2.16
	Mdn = 2.75	Mdn = 3.10	r = −0.23
Formal support	M = 15.36	M = 18.04	U = 658.50
	SD = 6.61	SD = 5.78	z = −1.92
	Mdn = 15.00	Mdn = 17.00	r = −0.21
Informal support	M = 10.94	M = 12.49	U = 737.00
	SD = 5.06	SD = 5.34	z = −1.22
	Mdn = 11.00	Mdn = 12.00	r = −0.13
Online support	M = 16.72	M = 15.30	U = 774.00
	SD = 8.85	SD = 8.14	z = −0.67
	Mdn = 14.50	Mdn = 14.00	r = −0.07
Offline support	M = 23.00	M = 26.79	U = 663.00
	SD = 9.14	SD = 8.34	z = −1.88
	Mdn = 24.00	Mdn = 25.00	r = −0.20
MSPSS Significant other	M = 3.73	M = 4.67	U = 708.00
	SD = 2.21	SD = 1.91	z = −1.95
	Mdn = 3.25	Mdn = 5.25	r = −0.21
MSPSS Family	M = 3.12	M = 3.64	U = 797.00
	SD = 1.79	SD = 1.99	z = −1.21
	Mdn = 3.00	Mdn = 3.75	r = −0.13
MSPSS Friend	M = 3.66	M = 4.04	U = 856.50
	SD = 1.87	SD = 1.64	z = −0.71
	Mdn = 3.75	Mdn = 4.25	r = −0.07
INQ-PB	M = 22.58	M = 19.11	U = 715.00
	SD = 10.23	SD = 10.04	z = −1.54
	Mdn = 22.00	Mdn = 18.00	r = −0.17
INQ-TB	M = 44.42	M = 40.78	U = 758.00
	SD = 13.52	SD = 12.72	z = −1.29
	Mdn = 45.00	Mdn = 42.00	r = −0.14

* Denotes significance at *p* < 0.05. SBQ-R = Suicide Behaviors Questionnaire – Revised. SIAS = Social Interaction Anxiety Scale. PHQ = Patient Health Questionnaire. SCS-R = Social Connectedness Scale-Revised. MSPSS = Multidimensional Scale of Perceived Social Support. INQ-PB = Interpersonal Needs Questionnaire-perceived burdensomeness. INQ-TB = Interpersonal Needs Questionnaire thwarted belongingness (TB).

**Table 4:** Descriptive statistics and MANOVA results for mood.

Sub-Scale	Suicide-Related Users (*n* = 33)	Non-Suicide Related Users (*n* = 57)	MANOVA Results
BRUMS Tension	M = 9.94	M = 7.88	F = 6.19
	SD = 3.50	SD = 3.95	*p* = 0.02
	Mdn = 13.00	Mdn = 7.00	d = 0.53
	T-score = 66	T-score = 64	
BRUMS Depression	M = 11.09	M = 7.81	F = 10.78
	SD = 4.49	SD = 4.62	*p* = 0.001
	Mdn = 12.00	Mdn = 8.00	d = 0.68
	T-score = 79	T-score = 68	
BRUMS Anger	M = 7.79	M = 6.21	F = 3.28
	SD = 4.53	SD = 3.63	*p* = 0.07
	Mdn = 8.00	Mdn = 6.00	d = 0.39
	T-score = 68	T-score = 63	
BRUMS Vigor	M = 3.55	M = 4.54	F = 2.74
	SD = 2.43	SD = 2.93	*p* = 0.10
	Mdn = 10.00	Mdn = 5.00	d = 0.36
	T-score = 40	T-score = 43	
BRUMS Fatigue	M = 11.85	M = 10.12	F = 3.64
	SD = 3.98	SD = 4.22	*p* = 0.06
	Mdn = 13.00	Mdn = 11.00	D = 0.41
	T-score = 66	T-score = 63	
BRUMS Confusion	M = 9.48	M = 6.86	F = 8.81
	SD = 3.87	SD = 4.14	*p* = 0.004
	Mdn = 10.00	Mdn = 6.00	d = 0.62
	T-score = 72	T-score = 63	

Note. Multivariate statistics: Wilks’ *Λ* (6,83) = 0.876, *p* = 0.08, partial *ƞ*^2^ = 0.124. BRUMS = Brunel Mood Scale.

## Data Availability

The data presented in this study are available on request from the corresponding author due to ethical approval requirements.

## Abbreviations

The following abbreviations are used in this manuscript:

BRUMS	Brunel Mood Scale
GHSQ	General Help-Seeking Questionnaire
H	Research hypothesis
INQ	Interpersonal Needs Questionnaire
MHP	Mental health practitioner
MSPSS	Multidimensional Scale of Perceived Social Support
PB	Perceived burdensomeness
PHQ-9	Patient Health Questionnaire
PIS	Participant Information Sheet
SIAS	Social Interaction Anxiety Scale
SBQ-R	Suicide Behaviors Questionnaire-Revised
SCS-R	Social Connectedness Scale-Revised
STBs	Suicidal thoughts/behaviors
TB	Thwarted belongingness
